# Predictors of survival in advanced oral cancers after salvage surgery with free tissue flap reconstruction

**DOI:** 10.1007/s00405-023-07888-z

**Published:** 2023-03-10

**Authors:** Anna Hafström, Peter Wahlberg, Stina Klasson, Lennart Greiff, Johanna Sjövall

**Affiliations:** 1 Department of ORL, Head and Neck Surgery, Skåne University Hospital, 221 85 Lund, Sweden; 2 Department of Clinical Sciences, Lund University, Lund, Sweden; 3 Department of Plastic Surgery, Skåne University Hospital, Lund, Sweden

**Keywords:** Head and neck cancer, Squamous cell carcinoma, Oral cavity, Prognosis, Extranodal extension, Free flap surgery

## Abstract

**Objective:**

To identify prognostic factors for patients with advanced persistent, recurrent, or 2nd primary oral cavity squamous cell carcinoma (OCSCC) potentially unsuitable for salvage surgery with free tissue flap (FTF) reconstruction.

**Materials and methods:**

A population-based cohort of 83 consecutive patients with advanced OCSCC who underwent salvage surgery with FTF reconstruction at a tertiary referral centre between 1990 and 2017. Retrospective uni- and multivariable analyses were performed to identify factors affecting all-cause mortality (ACM), i.e., overall survival (OS), as well as disease-specific mortality (DSM), i.e., disease-specific survival (DSS) after salvage surgery.

**Results:**

Median disease-free interval until recurrence was 15 months with recurrent stage I/II in 31% and III/IV in 69%. Median age at salvage surgery was 67 years (range 31–87) and the median follow-up (alive patients) 126 months. At 2, 5, and 10 years after salvage surgery, respectively, DSS rates were 61%, 44%, and 37% and OS rates 52%, 30%, and 22%. Median DSS was 26 and OS 43 months. Multivariable analysis identified recurrent clinical regional (cN-plus) disease [HR 3.57; *p* < .001] and elevated gamma-glutamyl transferase (GGT) [HR 3.30; *p* = .003] as independent pre-salvage predictors for poor OS after salvage, whereas initial cN-plus [HR 2.07; *p* = .039] and recurrent cN-plus disease [HR 5.14; *p* < .001] predicted poor DSS. Among post-salvage factors, extranodal extension according to histopathology [HR ACM 6.11; HR DSM 9.99; *p* < .001] as well as positive [HR ACM 4.98; DSM 7.51; *p* < 0.001] and narrow surgical margins [HR ACM 2.12; DSM HR 2.80; *p* < 0.01] emerged as independent factors for poor survival.

**Conclusion:**

While salvage surgery with FTF reconstruction is the primary curative option for patients with advanced recurrent OCSCC, the present findings may help guide discussions with patients who have advanced recurrent regional disease and high GGT preoperatively, especially if there is a small chance of reaching surgical radicality.

## Background

Head and neck squamous cell carcinoma (SCC) is the 6th most common cancer globally with the oral cavity as the most common site [1]. In early-stage disease, oral cavity SCC (OCSCC) is usually managed by surgery alone, while more advanced stages require multimodality treatment, e.g., surgery followed by adjuvant radiotherapy (RT). Despite curative intent, the recurrence rate is 15–45% depending on stage [2–10], and local or loco-regional failures account for about 90% of the recurrences [6, 9].

The 5-year overall survival (OS) for advanced recurrent tumors in the oral cavity after salvage surgery is reportedly between 18 and 45% [3, 4, 7, 8, 11–13]. The usual curative option for patients with advanced persistent, recurrent, or 2nd primary tumors in the oral cavity is salvage surgery in combination with free tissue flap (FTF) reconstruction, which may be combined with post-operative re-irradiation [14]. Despite advances in reconstructive FTF techniques, this procedure can cause significant complications resulting in substantial morbidities, including flap failure, dysphagia, dysarthria, osteoradionecrosis, and disfigurement as well as impaired quality of life [12, 15–19]. Thus, in terms of improving patient survival, it is important to assess who may or may not mostly benefit from the treatment. In this context, general factors to consider are the history of prior treatment, comorbidity, pre-salvage performance status, and extent of tumor (i.e., resectability) as well as patient cooperation and volition.

Specific factors associated with poor salvage surgery outcome for head and neck SCC include lack of disease-free interval (DFI) following previous definitive RT, advanced initial and recurrent tumor stage, presence of concomitant recurrent regional neck disease, and positive surgical margins [12, 20]. Focusing on OCSCC, Sun et al*.* reported primary TNM stage before initial treatment, extent of recurrence, and recurrent tumor size as significant independent prognostic factors for survival in a cohort of 81 patients [21]. In a cohort of 73 patients (FTF reconstruction in 42%), Chung et al., indicated that a short DFI (less than 8 months) until recurrence remained as a significant independent prognostic factor for disease-free survival (DFS), whereas recurrence in a previously treated field remained a factor for OS after salvage surgery [22]. According to Tam et al., the most important independent predictor for short OS after salvage was whether or not adjuvant radiotherapy (RT) or chemoradiotherapy (ChRT) had been administered following primary surgery in a cohort of 39 patients who underwent salvage surgery (FTF reconstruction in 68%) for local, regional, or loco-regional recurrences [8]. Being older than 62 years was found to be the second most important independent predictor. Goto et al. explored several prognostic factors for survival after salvage surgery in 69 patients with tongue SCC and reported that only extra nodal extension (ENE) remained as an independent prognostic factor for OS according to the multivariable analyses [23].

There is, however, a need to identify and better understand pre-operative factors to improve the care of patients who are candidates for salvage surgery with FTF reconstruction. Accordingly, the present study focused on a well-defined population-based cohort of consecutive patients with a difficult-to-treat problem, i.e., advanced persistent, recurrent, or 2nd primary tumors in the oral cavity. We determined survival and assessed features associated with outcome after such treatment, specifically in relation to OS and disease-specific survival (DSS) after salvage surgery.

## Methods

### Study design and population

The study was retrospective and involved all consecutive patients from a population-based cohort of 1.9 million inhabitants of the Southern Swedish Health Care Region who underwent salvage surgery with FTF reconstruction in the oral cavity between 1990 and 2017. Patients were either diagnosed at our tertiary Head and Neck Centre for Southern Sweden (an academic referral centre) or referred to us from any of the five sub-regional hospitals. This reflects that all treatment with curative intent for OCSCC in the region is performed at our centre. Approval for the study was granted by the Swedish Ethical Review Authority (ref. no. 2021–04447). Patients were identified and selected from our head and neck FTF reconstruction database (started in 1989; now containing > 600 patients).

Inclusion criteria were age ≥ 18 years, resectable advanced local or loco-regional failure or an advanced 2nd primary tumor in the oral cavity, treatment with curative intent, salvage surgery with wide field resection, squamous cell carcinoma, and FTF reconstruction. An advanced 2nd primary tumor was defined as an out-of-field recurrence and/or a DFI > 60 months that required FTF reconstruction. Patients with positive surgical margins who required a second surgery as part of their initial primary treatment were not included. Relevant data were collected from medical records. Demographics were analysed as well as data on histopathology, stage, and treatment of the primary and current tumors. If necessary, patients were retrospectively restaged according to the 7th Edition of the American Joint Committee on Cancer (AJCC)/Union for International Cancer Control (UICC): TNM classification of malignant tumours [24].

One to two weeks prior to the salvage surgery, WHO performance status, body mass index (BMI), and laboratory tests (haematology, nutritional, renal, and hepatic) were retrieved. BMI was defined as underweight (BMI ≤ 18.5), normal (BMI 18.6–24.9), overweight (BMI 25.0–29.9), or obese (BMI ≥ 30.0). Data on patient-reported alcohol over-consumption defined by the patient as “giving them problems” and smoking habits were collected as were data on comorbidities. The Charlson Comorbidity Index (CCI) was calculated [25].

### Follow-up data

Patients had clinical follow-ups every 3–6 months for at least 5 years until death or until June 1st, 2022. Date, localization, stage of any recurrence, and cause of death were recorded. Median follow-up time was assessed using the reversed Kaplan–Meier method. DFI was measured from the end of treatment of the primary tumor to diagnosis of the recurrent OCSCC. A persistent tumor was defined as a DFI of < 6 months. DFS after salvage surgery was measured from salvage surgery to recurrence or death by any cause. OS was assessed from salvage surgery to last follow-up visit or death, whereas DSS was measured from salvage surgery to last follow-up or dead of disease (DOD). Surviving patients still recurrence-free and/or alive at their last follow-up were censored on that date.

### Statistical analysis

Statistical analysis was performed using SPSS Statistics, version 25.0. Categorical variables were summarized as frequencies and percentages. Continuous variables were indicated as means and standard deviations or medians and ranges. Differences in OS and DSS after salvage between groups were analysed using the Kaplan–Meier method and the differences between survival curves were assessed by the log-rank test. Analyses of risk factors for all-cause mortality (ACM) and disease-specific mortality (DSM) after salvage surgery were performed using Cox proportional hazard analysis. Statistically significant predictors of OS (i.e., ACM) or DSS (i.e., DSM) after salvage (*p* < 0.05) in univariable analyses were introduced in multivariable analyses after considering proportional hazard, collinearity, and overfitting of the models.

## Results

### Primary tumors and treatment

Eighty-three patients met the inclusion criteria. Demographical and pre-salvage clinical data available are indicated in Tables 1 and 2. Median age at diagnosis of the primary tumors was 64 years (mean 61.7 ± 11.6, range 30–81). The most common localizations were tongue (41%), floor of mouth (FoM) (17%), lower alveolar list (13%), and buccal mucosa (12%), together representing 83% of the subsites. Clinical stages were I–II in 47 cases (57%) and III–IV in 36 (43%), and 80% of the patients had cN0-disease. Initial treatment had been multimodality therapy in 54% of the cases (surgery combined with RT in 46% and ChRT in 8%) and single modality therapy with surgery in 17%, RT in 24%, and ChRT in 5%. In all, 86% of the patients had earlier RT, 13% ChRT, and 71% (*n* = 59) surgery (whereof 39 had neck dissections and 6 also FTF reconstructions as part of the primary treatment).

**Table 1:** Pre-salvage factors predicting survival after salvage surgery with FTF reconstruction in the oral cavity according to Cox univariable regression analyses

		Events ACM	Univariable Cox ACM			Events DSM	Univariable Cox DSM		
Category	*n* (%)	*n* (%)	*p *value	HR	(95% CI)	*n* (%)	*p* value	HR	(95% CI)
Sex									
Female	37 (45)	31 (78)		Ref = 1		24 (65)		Ref = 1	
Male	46 (55)	36 (84)	0.754	1.08	0.67–1.75	21 (46)	0.832	1.06	0.59–1.92
Initial TNM-stage									
I	15 (18)	10 (67)	0.314	Ref = 1		8 (53)	0.387	Ref = 1	
II	32 (39)	25 (78)	0.346	1.42	0.68–2.97	16 (50)	0.769	1.14	0.49–2.66
III	14 (17)	12 (86)	0.302	1.56	0.67–3.62	6 (43)	0.887	0.93	0.32–2.67
IV	22 (26)	20 (91)	0.067	2.04	0.95–4.36	15 (68)	0.182	1.80	0.76–4.24
Initial cT stage									
T0^d^	3 (4)	2 (67)	0.945	Ref = 1		1 (33)	0.926	Ref = 1	
T1	18 (22)	14 (78)	0.974	0.83	0.19–3.68	10 (56)	0.803	1.30	0.17–10.16
T2	41 (49)	33 (80)	0.713	1.09	0.26–4.57	23 (56)	0.621	1.66	0.22–12.29
T3	11 (13)	9 (82)	0.760	1.05	0.23–4.89	6 (55)	0.716	1.48	0.18–12.34
T4a	10 (12)	9 (90)	0.871	0.98	0.21–4.53	5 (50)	0.886	1.17	0.14–10.03
Initial regional disease									
cN0	66 (80)	51 (77)		Ref = 1		32 (48)		Ref = 1	
cN+	17 (20)	16 (94)	0.**008**	2.18	1.23–3.86	13 (76)	**0.006**	2.52	1.31–4.83
Previous primary treatment									
RT ± Chemo	24 (29)	19 (79)	0.441	Ref = 1		14 (58)	0.574	Ref = 1	
Surgery only	14 (17)	9 (64)	0.242	0.62	0.28–1.37	7 (50)	0.324	0.64	0.26–1.56
Multimodal^a^	45 (54)	39 (87)	0.948	0.98	0.57–1.69	24(53)	0.445	0.78	0.40–1.49
DFI									
≥ 6 months	57 (69)	45 (79)		Ref = 1		19 (34)		Ref = 1	
< 6 months	26 (31)	22 (85)	**0.016**	1.89	1.13–3.18	26 (96)	**0.002**	2.63	1.45–4.77
Recurrence subsite^b^									
Tongue	27 (33)	23 (85)	0.344	Ref = 1		17 (63)	0.229	Ref = 1	
Alveolar list	22 (27)	18 (82)	0.175	0.65	0.35–1.21	9 (41)	0.055	0.45	0.20–1.02
FoM	20 (24)	16 (80)	0.333	0.73	0.38–1.38	11 (55)	0.407	0.72	0.34–1.55
Bucca	11 (13)	7 (64)	0.114	0.50	0.22–1.18	5 (45)	0.188	0.51	0.19–1.39
Recurrent TNM stage									
I	6 (7)	3 (50)	0.317	Ref = 1		2 (33)	0.459	Ref = 1	
II	20 (24)	16 (80)	0.157	2.44	0.71–8.40	10 (50)	0.322	2.16	0.47–9.87
II	10 (12)	8 (80)	0.157	2.61	0.69–9.89	7 (70)	0.150	3.18	0.66–15.3
IV^c^	47 (57)	40 (85)	0.068	2.99	0.92–9.69	26 (55)	0.161	2.80	0.66–11.8
Recurrent cT-category									
T0^d^	1 (1)	1 (100)		NI		1 (100)		NI	
T1	9 (11)	6 (67)	0.396	Ref = 1		4 (44)	**0.038**	Ref = 1	
T2	22 (27)	18 (82)	0.136	1.49	0.59–3.77	12 (55)	0.470	1.52	0.49–4.72
T3	16 (19)	14 (88)	0.608	2.08	0.80–5.43	13 (81)	0.051	3.06	0.99–9.42
T4a	35 (42)	28 (80)	0.369	1.26	0.52–3.05	15 (43)	0.871	1.10	0.36–3.31
Recurrent regional disease									
cN0	61 (73)	45 (74)		Ref = 1		27 (44)		Ref = 1	
cN+	22 (27)	22 (100)	** < 0.001**	4.87	2.79–8.49	18 (82)	** < 0.001**	5.65	2.97–10.8
Recurrent cN-category									
N0	61 (73)	45 (74)	** < 0.001**	Ref = 1		27 (44)	** < 0.001**	Ref = 1	
N1	4 (5)	4 (100)	**0.007**	4.31	1.49–12.5	3 (75)	**0.020**	4.29	1.25–14.7
N2	16 (19)	16 (100)	** < 0.001**	4.67	2.54–8.60	13 (81)	** < 0.001**	5.56	2.76–11.2
N3	2 (3)	2 (100)	** < 0.001**	32.6	6.28–169	2 (100)	** < 0.001**	35.6	6.73–187

Numbers in bold indicate statistically significant values

*Cox* Cox regression analyses, *HR* hazard ratio, *ACM* all-cause mortality, *DSM* disease-specific mortality, *CI* confidence interval, *NI* not included in the analyses, *DFI* disease-free interval, *FoM* floor of mouth, *RT* radiotherapy, *Chemo* chemotherapy (concomitant), *Ref* reference, *NI* not included in the analyses

^a^Multimodality treatment was surgery followed by postoperative radiotherapy in 45% and chemoradiotherapy in 8%

^b^Two oral subsites were not included in the univariable analyses: lip (*n* = 2) and hard palate (*n* = 1)

^c^Two of the 47 patients with stage IV disease had advanced recurrent nodal metastases (cN3) and thus stage IV B while the other 45 had stage IV A disease

^d^One patient with a primary tumor of the hard palate (T2N2c) was not included in the univariable analyses because of very advanced regional recurrence with extensive tumor growth into the FoM (yT0RN3)

**Table 2:** Pre-salvage factors predicting survival and risk for death after salvage surgery with FTF reconstruction in the oral cavity according to Cox univariable regression analyses (step wise backward)

		Events ACM	Univariable Cox ACM			Events DSM	Univariable Cox DSM		
Category	*n* (%)	*n* (%)	*p* value	HR	(95% CI)	*n* (%)	*p* value	HR	(95% CI)
BMI^a^									
Underweight	7 (9)	7 (100)	0.232	Ref = 1		6 (86)	0.486	Ref = 1	
Normal	52 (63)	44 (85)	0.893	0.95	0.42–2.11	29 (56)	0.528	0.75	0.31–1.82
Overweight	14 (17)	8 (57)	0.111	0.44	0.16–1.21	6 (43)	0.134	0.42	0.13–1.31
Obese	9 (11)	7 (78)	0.954	0.97	0.34–2.78	4 (44)	0.561	0.69	0.19–2.44
Reported alcohol overconsumption									
No	40 (48)	34 (85)	0.360	Ref = 1		24 (60)	0.555	Ref = 1	
Yes	16 (19)	13 (81)	0.973	0.99	0.52–1.89	7 (44)	0.620	0.81	0.35–1.88
Not known	27 (33)	20 (74)	0.172	0.68	0.39–1.18	14 (52)	0.286	0.70	0.36–1.35
Smoking									
No	23 (28)	19 (83)	0.238	Ref = 1		15 (65)	0.158	Ref = 1	
Current smoker	29 (35)	25 (86)	0.611	0.79	0.31–1.98	15 (52)	0.901	0.96	0.47–1.96
Ex-smoker	25 (30)	17 (68)	0.925	1.04	0.43–2.56	9 (36)	0.082	0.48	0.21–1.10
Not known	6 (7)	6 (100)	0.220	0.56	0.22–1.42	6 (100)	0.419	1.48	0.57–3.83
Comorbidity									
CCI 0–2	36 (43)	26 (72)		Ref = 1		20 (56)		Ref = 1	
CCI ≥ 3	47 (57)	41 (87)	0.371	1.26	0.76–2.07	25 (53)	*0.773*	1.09	0.60–1.97
Cardiovascular disease									
No	54 (65)	43 (80)		Ref = 1		31 (57)		Ref = 1	
Yes	29 (35)	24 (83)	0.748	1.09	0.66–1.79	14 (48)	0.648	0.86	0.46–1.62
Hypertension									
No	54 (65)	44 (81)		Ref = 1		28 (52)		Ref = 1	
Yes	29 (35)	23 (79)	0.947	1.02	0.61–1.69	17 (59)	0.723	1.12	0.61–2.04
Diabetes									
No	72 (87)	59 (82)		Ref = 1		38 (53)		Ref = 1	
Yes	11 (13)	8 (73)	0.995	1.00	0.48–2.09	7 (64)	0.506	1.32	0.59–2.95
COPD									
No	70 (84)	56 (80)		Ref = 1		38 (54)		Ref = 1	
Yes	13 (16)	11 (85)	0.484	1.26	0.66–2.41	7 (54)	0.783	1.12	0.50–2.51
Anaemia^b^									
No	72 (89)	57 (79)		Ref = 1		37 (51)		Ref = 1	
Yes	9 (11)	9 (100)	0.094	1.83	0.90–3.72	7 (78)	0.080	2.07	0.92–4.66
Hypoalbuminemia^b^									
No	62 (77)	49 (79)		Ref = 1		33 (53)		Ref = 1	
Yes	19 (23)	18 (95)	0.088	1.60	0.93–2.75	12 (63)	0.176	1.58	0.81–3.07
Pathologically elevated ALP^b^									
No	55 (68)	43 (78)		Ref = 1		30 (55)		Ref = 1	
Yes	26 (32)	24 (92)	0.246	1.35	0.81–1.23	15 (58)	0.519	1.23	0.66–2.28
Pathologically elevated GGT^b^									
No	69 (85)	55 (80)		Ref = 1		37 (54)		Ref = 1	
Yes	12 (15)	12 (100)	** < 0.001**	4.67	2.31–9.43	8 (67)	**0.002**	3.61	1.57–8.27

Numbers in bold indicate statistically significant values

*HR* hazard ratios, *CI* confidence interval, *ACM* all-cause mortality, *DSM* disease-specific mortality, *Ref* reference, *BMI* body mass index, *CCI* Charlson comorbidity index, *COPD* chronic obstructive pulmonary disease, *ALP* alkaline phosphatase, *GGT* gamma-glutamyl transferase

^a^Underweight defined as BMI ≤ 18.5, normal as BMI 18.6–24.9, overweight as BMI 25.0–29.9, and obese as BMI ≥ 30.0

^b^According to pre-salvage surgery blood workup within 2 weeks of the FTF salvage surgery. For definition of cut-off points, please see the main text

### Recurrent (i.e., persistent, recurrent, or advanced 2nd primary) tumors and treatment

**Fig. 1:**
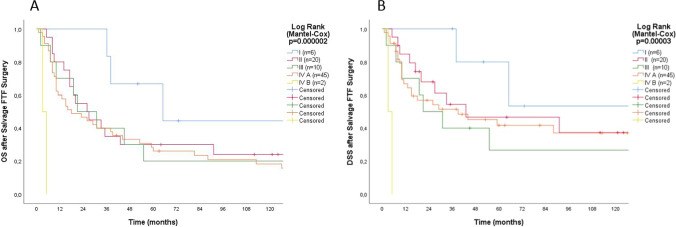
**A** Overall survival (OS) and **B** disease-specific survival (DSS) for 83 patients after salvage surgery with FTF reconstruction in the oral cavity according to clinical TNM stage of the recurrences

**Fig. 2:**
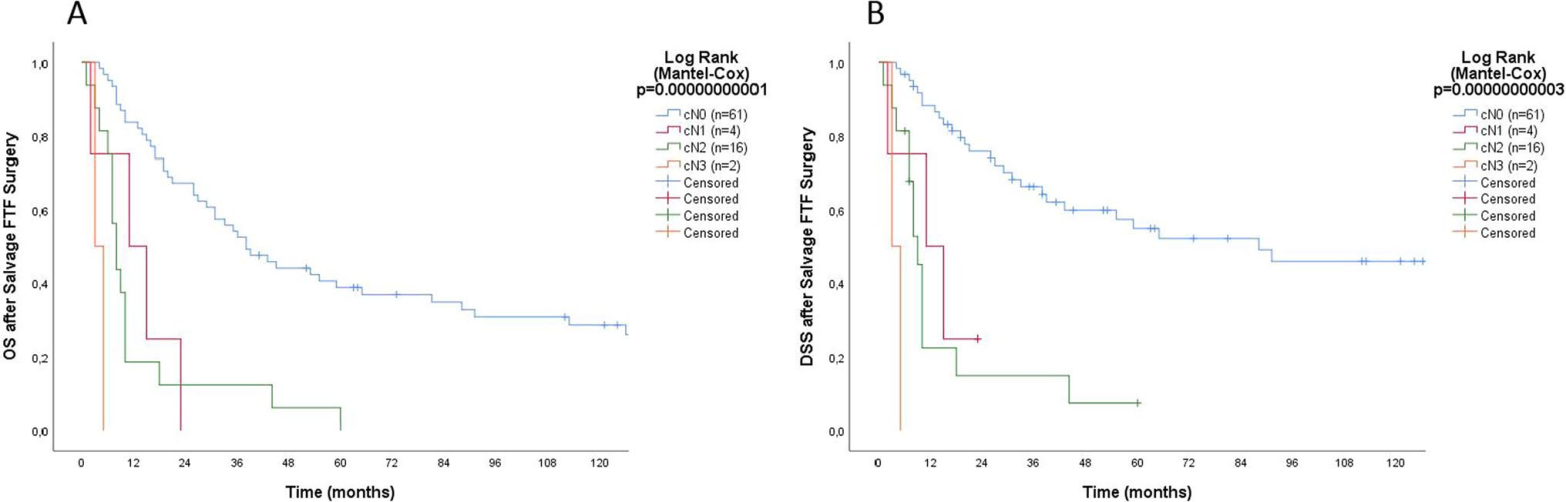
**A** Overall survival (OS) and **B** disease-specific survival (DSS) for 83 patients after salvage surgery with FTF reconstruction in the oral cavity according to clinical N-stage (cN) of the recurrences

Median DFI until diagnosis of the condition that resulted in salvage FTF surgery was 15 months (range 0–216). After the end of primary treatment, 27 patients (32%) were diagnosed with persistent disease, 45% (*n* = 37) with recurrent disease within 5 years, and 23% (*n* = 19) after 5 years. Of the 27 patients with persistent disease, 59% had initial definitive RT, 11% ChRT, 7% surgery (i.e., two cases, both with histopathological negative tumor margins), and 22% multimodal treatment (surgery with adjuvant RT in 15% and ChRT in 7%). The recurrences were classified as stage I-II disease in 26 cases (31%) and III-IV in 57 (69%) (Fig. 1). The oral subsites were tongue (33%), lower alveolar list (27%), and FoM (24%). Pure local recurrences (cN0-disease) were diagnosed in 73%, whereas the remainder also presented regional recurrences (cN-plus) (Table 1, Fig. 2). Eight of the 83 patients (10%) were considered having advanced 2nd primary tumors. Two of these patients had developed new SCC in oral cavity subsites not adjacent to the initial tumor sites (out of field recurrences) within the 5 years follow-up after end of treatment for their primary tumors. The other six cases were diagnosed with a new OCSCC after the 5 years follow up had ended and had a median DFI of 118 (mean 127) months].

Median age at salvage surgery was 67 years (mean 65.5 ± 11.4, range 31–87). The performance status was WHO 0 in 96% of the cases (the remainder had WHO 1) and 43% had CCI 2 or less. In terms of BMI, 9% were considered underweight, 17% overweight, and 11% obese, while 63% had normal BMI (Table 2). At salvage surgery, 19% reported alcohol overconsumption and 35% were current smokers. The pre-operative blood work indicated anaemia (Hb < 117 g/L) in 11%, hypoalbuminemia (< 36 g/L) in 23%, pathologically elevated alkaline phosphatase (ALP > 1.9 ukat/L) in 32%, and pathologically elevated gamma-glutamyl transferase (GGT > 1.9 ukat/L) in 15% (Table 2).

Most salvage resections were performed by three head and neck surgeons who took part as chief or assistant surgeons in 87% of the cases. The mobile tongue was resected in 48% of the cases, FoM in 63%, marginal mandibular resections in 15% and segmental in 42%. All patients had neck dissections (selective supra omohyoid in 60%, radical in 19%, and modified radical in 21% which were performed bilaterally in half of these cases). Reconstructions comprised fasciocutaneous radial forearm FTFs in 67%, osteocutaneous fibular in 20%, osteocutaneous radial forearm in 5%, fasciocutaneous anterolateral thigh (ALT) in 5%, rectus abdominis and gracilis muscle in 1% each. One patient had both a fibular and an ALT flap. During the first two postoperative weeks, 13 patients (16%) were readmitted to the operating room due to bleeding and/or compromised flaps. The FTFs were salvaged in 7 cases, new FTFs were established in 3, and regional flaps in 3 (indicating a 93% success rate of primary flap establishment). There was no perioperative mortality. According to the histopathology reports, surgical margins were negative in 43%, narrow (< 5 mm) in 46%, and positive in 11% of the cases. Perineural or perivascular tumor growth was evident in 27%. ENE according to the histopathology reports was observed in 23% (Table 3), i.e., in both patients with recurrent cN3 disease, 81% with cN2-disease, 50% with cN1-disease, and 3% with cN0-disease. Adjuvant RT following salvage surgery was administered to 35% of the 83 patients (Table 3).

**Table 3:** Post-salvage surgery factors predicting survival and risk for death after salvage surgery with FTF reconstruction in the oral cavity according to Cox univariable regression analyses

		Events ACM	Univariable Cox ACM			Events DSM	Univariable Cox DSM		
Category	*n* (%)	*n* (%)	*p* value	HR	(95% CI)	*n* (%)	*p* value	HR	(95% CI)
Histopathological margins									
Negative	36 (43)	25 (69)	** < 0.001**	Ref = 1		11 (31)	** < .001**	Ref = 1	
Narrow^a^	38 (46)	33 (87)	**0.011**	1.98	1.17–3.35	25 (66)	**0.001**	3.20	1.57–6.54
Positive	9 (11)	9 (100)	** < 0.001**	5.28	2.41–11.5	9 (100)	** < 0.001**	10.7	4.33–26.3
Extranodal extension									
No	64 (77)	48 (75)		Ref = 1		28 (44)		Ref = 1	
Yes	19 (23)	19 (100)	** < 0.001**	4.32	2.44–7.63	17 (89)	** < 0.001**	5.78	3.04–11.0
Perineural or perivascular invasion									
No	61 (73)	49 (80)		Ref = 1		29 (48)		Ref = 1	
Yes	22 (27)	18 (82)	0.238	1.39	0.80–2.40	16 (73)	**0.033**	1.95	1.05–3.60
Acute re-operation									
No	70 (84)	56 (80)	0.992	Ref = 1		37 (53)	0.964	Ref = 1	
Bleeding	6 (7)	5 (83)	0.985	1.01	0.40–2.52	4 (67)	0.789	1.15	0.41–3.23
Compromised flap	7 (8)	6 (86)	0.898	1.06	0.45–2.46	4 (57)	0.946	1.04	0.37–2.91
Post-op infection									
No	64 (77)	48 (75)		Ref = 1		32 (50)		Ref = 1	
Yes	19 (23)	19 (100)	**0.016**	1.96	1.13–3.39	13 (68)	0.071	1.83	0.95–3.52
Post op fistula									
No	65 (78)	49 (75)		Ref = 1		33 (51)		Ref = 1	
Yes	18 (22)	18 (100)	0.078	1.64	0.95–2.84	12 (67)	0.247	1.48	0.76–2.89
Adjuvant RT post-salvage surgery									
No	54 (65)	44 (81)		Ref = 1		26 (48)		Ref = 1	
Yes	29 (35)	23 (79)	0.656	1.12	0.67–1.87	19 (66)	0.213	1.46	0.80–2.65

Numbers in bold indicate statistically significant values

*Cox* Cox regression analyses, *HR* hazard ratio, *CI* confidence interval, *ACM* all-cause mortality, *DSM* disease-specific mortality, *RT* radiotherapy, *Ref* reference

^a^Defined as margins < 5 mm

### Follow-up and survival after salvage surgery with FTF reconstruction

Median follow-up after salvage surgery for patients who were still alive was 137 months (range 49–254). During follow-up, 48 patients (58%) were diagnosed with new recurrences, evenly distributed between a local recurrence (*n* = 16), a regional or loco-regional recurrence (*n* = 16), and a recurrence that included distant metastases (*n* = 16) (7 only distant, 2 local-distant, 3 regional-distant, and 4 with loco-regional plus distant disease). Median DFS after salvage was 26 months [95% CI 11.7–40.3] with DFS rates of 52.6 ± 5.6% at 2 years, 41.9 ± 5.8% at 5 years, and 35.6 ± 5.9% at 10 years.

During follow-up, 81% (*n* = 67) of the cohort died, whereof 54% (*n* = 45) were considered dead of disease (DOD). New primary cancers were diagnosed in 13% (*n* = 11), two patients with lung cancers, one oesophageal cancer, two rectal cancers, and six new head and neck cancers, i.e., 2nd or 3rd primary tumors. Median DSS after salvage was 43 months [95% CI 16.1–69.9] with DSS rates of 60.6 ± 5.5% at 2 years, 43.5 ± 5.9% at 5 years, and 36.9 ± 6.1% at 10 years**.** Median OS after salvage was 26 months [95% CI 14.1–37.9] with OS rates of 51.8 ± 5.5% at 2 years, 30.1 ± 5.0% at 5 years, and 21.7 ± 4.7% at 10 years.

The 61 patients who underwent salvage surgery due to local recurrences had significantly better survival (2 years OS 67.2% and 5 years 39.3%) than the 22 patients with loco-regional recurrent (cN-plus) disease (2 years OS 9.1% and 5 years 4.5%) (*p* < 0.001). As seen in Fig. 2, the two patients with recurrent cN3 disease died within 6 months after the salvage surgery, whereas 25% with recurrent cN2 and 25% with cN1 died within 6 months. Only two of the 22 (9.1%) patients with loco-regional recurrences (cN-plus disease) survived more than 2 years after salvage surgery.

Eight patients (50% male) survived < 6 months after salvage surgery. Six of them (75%) were older than 70 years *cf.* 31% in the rest of cohort (*p* = 0.019). Recurrent cN-plus disease was more common for the short survival group (75%) *cf*. the other patients (21%) (*p* = 0.004). All 6 patients with recurrent cN-plus disease and very short survival had ENE according to salvage surgery histopathology. ENE was more common (75%) than in the rest of the cohort (17%) (*p* = 0.001), and more patients had positive surgical margins (50%) than the other patients (7%) (*p* = 0.004). All patients with short survival had received RT before and two received re-irradiation after the salvage surgery. None of the patients with very short survival died because of peri- or post-operative complications. Instead, all 8 succumbed to their disease (DOD) after being diagnosed with early recurrences (4 loco-regional, 2 regional-distant, and 2 distant disease).

### Pre-salvage factors

Pre-operative factors that had a statistically significant impact on survival after salvage (ACM and DSM) were first identified by univariable analyses as depicted in Tables 1 and 2. Shorter DFI (in 1 month increments) was a significant predictor for OS [HR ACM 0.99 (95% CI 0.99–1.00) *p* = 0.035)] and DSS [HR DSM 0.99 (95% CI 0.98–1.00; *p* = 0.028)] after salvage. Age at salvage surgery (in 1 year increments) had no significant impact on OS or DSS (*p* > 0.05). Based on multivariable analysis, recurrent regional disease (cN-plus) [HR ACM 3.57 (95% CI 1.97–6.46) *p* < 0.001] and elevated GGT [HR ACM 3.30 (95% CI 1.51–7.23) *p* = 0.003] remained as independent pre-operative predictors for short OS after salvage after adjusting for cN-plus of the primary, age at salvage (1 year increments), and DFI < 6 months. Initial regional disease (cN-plus) [HR DSM 2.07 (95% CI 1.04–3.96) *p* = 0.039] and recurrent regional disease (cN-plus) [HR DSM 5.14 (95% CI 2.66–9.93 *p* < 0.001] remained as independent preoperative predictors for short DSS after adjusting for age at salvage (1 year increments), DFI < 6 months, and elevated GGT.

### Post-salvage factors

Results from the univariable analyses of post-salvage surgery factors predicting survival (ACM and DSM) are listed in Table 3. With multivariable analyses, ENE [HR 4.45 (95% CI 2.47–8.03 *p* < 0.001] as well as poor radicality with positive margins [HR 6.12 (95% CI 2.71–13.8) *p* =  < 0.001] and narrow margins [HR 2.03 (95% CI 1.19–3.46) *p* = 0.009] *cf*. free margins [HR 1.00 (i.e., reference) *p* < 0.001] and having a post-operative infection [HR 2.39 (95% CI 1.36–4.20) *p* = 0.002] remained as independent predictors for short OS (ACM) after adjusting for age at salvage (1 year increments). Poor radicality with positive [HR 10.5 (95% CI 4.13–26.7) *p* < 0.001] and narrow margins [HR 3.06 (95% CI 1.49–6.26) *p* = 0.002] *cf*. free margins according to histopathology [HR 1.00 (i.e., reference) *p* < 0.001] as well as ENE [HR 5.58 (95% CI 2.86–10.9) *p* < 0.001] remained as independent predictors for short DSS (DSM) after having adjusted for age at salvage (1 year increments) and perineural or perivascular invasion.

## Discussion

In this study, reflecting a well-defined population-based cohort treated at a tertiary academic referral center, we present survival data as well as potential predictors that appear to be associated with poor outcome for patients after salvage surgery  reconstructed with FTFs due to advanced persistent, recurrent, or 2nd primary tumors in the oral cavity. The survival data demonstrate OS rates of 52%, 30%, and 22%, respectively, at 2, 5, and 10 years after salvage surgery, and corresponding DSS rates of 61%, 44%, and 37%. Furthermore, median OS and DSS, respectively, was 26 and 43 months. The outcome is seemingly in accordance with previous reports on recurrent OCSCC. For example, Borsetto et al. reported a 5 years OS rate of 18% (median OS 17 months *cf.* 26 in our study) after salvage for 33 patients with recurrent OCSCC treated with curative intent [13]. In contrast, Tam et al*.* indicated a 43% 5 years OS in a cohort of 39 patients after undergoing salvage surgery due to local, regional, or loco-regional recurrent OCSCC [8]. Their patients were slightly younger than ours (median 64 *cf*. 67 years). Also, the seemingly better outcome in their study might be that the recurrent T-stage was lower (59% T1 and T2 *cf*. 30% T1 and T2 in our study) and that only 18% had recurrent regional metastases (*cf.* 27% in our study). Furthermore, FTF reconstruction was only performed in 68% of their cases, whereas it was an inclusion criterion in our study. Chung et al. reported a 5 years OS rate of 55% in a cohort of 73 OCSCC patients who received surgery-based salvage treatment [22]. However, the fact that FTF reconstruction was performed in only 42% of their cases might indicate less advanced T-stages in their cohort (*cf*. our study). Taken together, survival outcomes indicate that salvage surgery with FTF reconstruction is fully justifiable for patients with advanced persistent, recurrent, or 2nd primary tumors in the oral cavity.

For any factor to be useful as predictor of survival following salvage surgery with FTF reconstruction, it needs to be known when determining whether to recommend the salvage procedure to the patient. Accordingly, the factor can either concern information available at the primary diagnosis and treatment, or new information that has arisen when considering possible salvage treatment. In the former case, available data suggest advanced primary TNM-stage [13, 21], advanced initial N-stage [22], close or positive margins at primary resection [13], and adjuvant RT or ChRT administered following the primary surgery [8, 22] as negative predictors. In the latter case, age greater than 62 years [8, 26], advanced recurrent T-stage [21, 22, 26], presence of a loco-regional recurrent disease (lymph node metastasis) [2, 11, 22, 27, 28], and short DFI [(< 6 months) [27], (< 8 months) [22], (< 12 months) [11], and (< 18 months) [3, 7] as well as a recurrence within the previous treatment field *vs*. an out-of-field recurrence (48 vs. 85% OS rates) [22], have been reported as negative predictors for survival. Moreover, a moderate (vs. heavy or no) alcohol consumption has been associated with a significantly reduced risk of death by Borsetto et al. [13]. In our study, univariable analyses confirmed many of these negative pre-salvage predictors. However, the multivariable analysis suggests that of the factors known pre-salvage, only regional (cN-plus) disease at the primary diagnosis as well as detection of regional (cN-plus) disease in the salvage situation remained as independent significant predictors for DSM, whereas regional (cN-plus) disease and elevated GGT in the salvage situation remained for ACM.

Of the negative predictors for survival noted above, nodal status in the salvage situation may be of particular interest. Our findings confirmed that patients with a loco-regional persistent, recurrent, or advanced 2nd primary disease had an almost four times higher risk of death after salvage than patients with a local disease. Our findings are corroborated by Borsetto et al., who reported that patients with local recurrencies had significantly better survival rates (1 year OS 41%) than those with regional (1 year OS 20%) or loco-regional (1 year OS 14%) recurrences in a cohort of 83 individuals extracted from a reconstruction database where only 33 were treated with curative intent [13]. None of their patients with recurrences that included regional metastases survived more than 4 years. In our cohort, only two of the 22 patients with cN-plus disease at salvage were alive 2 years after salvage surgery and none after 5 years. In contrast to the study of Borsetto et al., which included 15 patients with only regional recurrent disease, in our study only one patient with a recurrent T0 N-plus disease was included. This tumor was, however, so advanced that it also involved the FoM and mandible. Moreover, Ord et al*.* indicated that no patients with loco-regional recurrences (*n* = 50) were salvaged in a cohort of 354 patients with OCSCC treated primarily by surgery with or without adjuvant therapy [2]. Similarly, Matsuura et al*.* demonstrated that a presence of lymph node metastasis was associated with poor OS in a cohort of 46 patients with local or loco-regional OCSCC relapses subjected to salvage surgery and where 78% received distant or regional flaps [28]. Despite a limited number of patients with recurrent cN3 disease in our study, our results agree with the notion that the more advanced the recurrent cN-stage, the greater the risk for short survival after salvage.

In addition to nodal status at salvage, elevated pre-salvage plasma GGT emerged as a predictor for poor survival and remained as an independent predictor for OS after salvage in our multivariable analysis. A cause of high GGT is alcohol over-consumption. Reported alcohol over-consumption did, however, not emerge as a predictor for poor survival in our study. However, data on alcohol consumption was available only for 67% of the patients, and such data might inherently be underestimated. Nevertheless, we found no other cause for the observed high GGT. For example, none of the patients were diagnosed with primary liver cancer or metastases to the liver. Furthermore, there was no correlation between GGT and high BMI, which is another known cause of high GGT. We suggest the possibility that alcohol over-consumption may be a predictor of poor survival after surgery with FTF reconstruction for persistent, recurrent, or 2nd primary tumors in the oral cavity, and propose that the level of phosphatidylethanol (PEth), a direct nonoxidative metabolite of ethanol [31], should be measured in all relevant patients.

In several studies, a short DFI prior to salvage has been associated with poor salvage surgery outcomes for head and neck cancer. These studies have, however, reported different cut-offs for short DFI, e.g., < 6 months [27], < 8 months [22], < 12 months [11], and < 18 months [3, 7]. A DFI of less than 6 months (the definition for persistent disease according to the Swedish National Head and Neck Cancer Register) was a significant predictor for both ACM and DSM according our univariable analyses. However, a short DFI was not a significant independent predictor for survival according to the multivariable analyses. In contrast, others have reported a lack of DFI following previous definitive RT as negative predictor of survival [12, 20]. Though 70% of our patients with a DFI < 6 months had initial definitive RT or ChRT, initial primary treatment modality was not a significant predictor of survival.

Second primary SCC in the oral cavity is a significant problem and have a negative impact on survival [35, 36]. Patients who suffer from local recurrences are, however, considered to have a poorer prognosis than those with 2nd primary tumors that more often can be treated by simpler means and with curative intent [36]. The distinction between locally recurrent tumors and 2nd primary tumors can be imperative for proper diagnosis and treatment [37]. Moreover, a misclassification of a 2nd primary tumor as a local recurrence or vice versa may represent a selection bias when attempting to compare study outcomes that focus on treatment effects and survival though no recognized definition exists for the two entities [37]. Only 9% of the patients in our cohort were considered as having advanced 2nd primary tumors as the reason for salvage surgery with FTF reconstruction due to either recurrences in subsites not adjacent to the initial tumors or after the 5 years follow-up. We found no significant differences in OS and DSS after salvage for patients with advanced 2nd primary tumors and recurrences in our study. Thus, our findings do not concur with Chung et al*.* who reported worse survival for recurrences within the previous treatment field vs. an out-of-field recurrence that might be interpreted as 2nd primary tumors (48 vs. 85% OS, respectively) [22]. Given that all patients in our study had salvage surgery with FTF reconstruction with curative intent, we did not consider the bias for survival outcome as suggested above [37, 38].

Of the variables that could be determined intra- or postoperatively, our multivariable analyses indicate that ENE of the recurrence (according to the pathology report) is a strong independent predictor of poor outcome—with more than four times higher risk for death (ACM) and five times for DSM. Concurring with our data, Goto et al*.*, who investigated 69 patients with recurrent OCSCC of the tongue subsite, reported that DFI, stage, nodal status, and ENE were significant prognostic indicators on univariable analysis, but that only ENE remained as an independent prognostic factor for OS in the multivariable analysis [23]. Regional nodal metastasis is a well-known major determinant of survival in patients with OCSCC, with size and number of nodal metastases as well as ENE being important characteristics that influence prognosis [29]. Accordingly, ENE has emerged as one of the most important prognostic factors in primary head and neck SCC, and clinical ENE is now included for OCSCC in the updated 8th edition of the AJCC/UICC staging criteria [30]. However, in a salvage situation, Borsetto et al., reported that pathological ENE of the primary tumor did not remain as an independent predictor for survival in their multivariable analyses [13]. Instead, they hypothesized that the poorer prognosis for patients with ENE co‐varied with loco-regional *cf.* only local recurrences. In our study, clinical ENE of the primary tumors could not be assessed. In contrast, post-salvage observations in our study support the importance of a regional metastasis (cN-plus) at the time of salvage surgery with FTF reconstruction for OCSCC. Taken together, and potentially reflecting the aggressiveness of a cancer, ENE is a key prognostic factor for head and neck cancer in general as well as in salvage situations. We suggest that nodal status and ENE (potentially evaluated by PET and CT/MRI), when advanced persistent, recurrent, or 2nd primary tumors in the oral cavity are detected, may be considered when determining whether to recommend salvage surgery with FTF reconstruction to these patients.

Radicality according to histopathology was another post-salvage factor that remained as an independent survival predictor. Accordingly, both positive and narrow histopathological margins independently predicted poor survival after salvage surgery with FTF reconstruction (*cf.* free margins). We acknowledge that no universal guidelines exist to define a margin as adequate or inadequate, and that different pathologists and surgeons adopt different criteria. Nonetheless, we chose to define narrow margins as < 5 mm [32]. Our results concur with the consensus that the distance between the neoplastic lesion and the resection edge is the best prognostic factor for local control [33, 34]. Moreover, our findings correspond with Matsuura et al*.* who reported that positive surgical margins at salvage were associated with a worse OS in a cohort of 46 patients (31% 5 years OS) with OCSCC recurrences treated with salvage surgery where 78% received a distant or regional flap [28].

There were limitations to our study. First, the number of cases was limited, which might pose a risk of overinterpreting the data. Data regarding the depth of invasion of the primary and recurrent tumors were lacking. Furthermore, while representing consecutive patients from a defined population-based cohort, the patients were selected as considered appropriate for curative salvage surgery with FTF reconstruction. Larger, multi-institutional, studies are preferable and warranted.

In conclusion, while salvage surgery with FTF reconstruction is the primary curative option for patients with advanced persistent, recurrent, or 2nd primary tumors in the oral cavity, our findings may help to guide discussions with patients with advanced recurrent regional disease and high GGT preoperatively, especially if there are clinical indications of ENE or a small chance of reaching radical surgical margins.

## Data Availability

The datasets generated during and/or analysed during the current study are not publicly available due to patient confidentality and Swedish law, but are available from the corresponding author on reasonable request.
